# Assessment of Temporary Medical Clinics During the Arbaeenia Mass Gathering at Al-Karkh, Baghdad, Iraq, in 2014: Cross-Sectional Study

**DOI:** 10.2196/10903

**Published:** 2019-09-26

**Authors:** Faris Lami, Ali Abdalkader Ali, Kareem Fathullah, Hana Abdullatif

**Affiliations:** 1 Department of Community and Family Medicine College of Medicine University of Baghdad Baghdad Iraq; 2 Directorate of Public Health Iraq Ministry of Health Baghdad Iraq; 3 Erbil Directorate of Health Iraq Ministry of Health Erbil Iraq

**Keywords:** medical staff, medical services, mass gathering, Iraq

## Abstract

**Background:**

During mass gatherings, public health services and other medical services should be planned to protect attendees and people living around the venue to minimize the risk of disease transmission. These services are essential components of adequate planning for mass gatherings. The Arbaeenia mass gathering signifies the remembrance of the death of Imam Hussain, celebrated by Shiite Muslims, and takes place in Karbala, which is a city in southern Iraq. This annual mass gathering is attended by millions of people from within and outside Iraq.

**Objective:**

This study aimed to map the availability of medical supplies, equipment, and instruments and the health workforce at the temporary clinics located in Al-Karkh, Baghdad, Iraq, in 2014.

**Methods:**

This assessment was conducted on the temporary clinics that served the masses walking from Baghdad to Karbala. These clinics were set up by governmental and nongovernmental organizations (NGOs) and some faith-based civil society organizations, locally known as mawakib. We developed a checklist to collect information on clinic location, affiliation, availability of safe water and electricity, health personnel, availability of basic medical equipment and instruments, drugs and other supplies, and average daily number of patients seen by the clinic.

**Results:**

A total of 30 temporary clinics were assessed: 18 clinics were set up by the Ministry of Health of Iraq and 12 by other governmental organizations and NGOs. The clinics were staffed by a total of 44 health care workers. The health workers served 16,205 persons per day, an average of 540 persons per clinic, and 368 persons per health care worker per day. The majority of clinics (63% [19/30]-100% [30/30]) had basic medical diagnostic equipment. Almost all clinics had symptom relief medications (87% [26/30]-100% [30/30]). Drugs for diabetes and hypertension were available in almost half of the clinics. The majority of clinics had personal hygiene supplies and environmental sanitation detergents (78%-90%), and approximately half of the clinics had medical waste disposal supplies. Instruments for cleansing and dressing wounds and injuries were available in almost all clinics (97%), but only 4 clinics had surgical sterilization instruments.

**Conclusions:**

Although temporary clinics were relatively equipped with basic medical supplies, equipment, and instruments for personal medical services, the health workforce was insufficient, given the number of individuals seeking care, and only limited public health service, personal infection control, and supplies were available at the clinics.

## Introduction

### Background

The assembly of large masses of people in a small space for a short period has a potential health risk to both the event attendees and the local population. Such risks vary depending on the number of attendees, nature of the gathering, extent of the space, and other environmental factors, such as infrastructure and level of preparedness. These events, known as mass gatherings (MGs), have several definitions [1,2]. The World Health Organization (WHO) defines MGs as “events attended by a number of people sufficient to strain the planning and response resources of a community, state or nation” [3].

Masses gather for many reasons including religious activities, festivals, sporting events, political rallies, and other purposes. The most common religious MGs in the Middle East are the Hajj at Mecca in Saudi Arabia and the Arbaeenia in Karbala for the remembrance of Imam Hussain, celebrated by Shiite Muslims [4]. Both events occur annually.

Previous researchers had assessed the risks of communicable diseases that are associated with MGs and the risks for mortality and morbidity during MGs [4-6]. Moreover, they outlined approaches to risk assessment and mitigation, drew attention to some key challenges encountered by organizers and participants, identified most efficient public health interventions, and identified the need for robust research into health risks for noncommunicable diseases during MGs [4-7].

Infectious disease outbreaks and mass causalities have been reported during the Hajj and other MGs [8,9]. The risk of spreading infectious diseases to host countries and to other parts of the world has tremendous global health security implications and is of particular concern during MGs. In MGs, the risk of infectious diseases increases because the participants might be the source of infection or they are susceptible to infectious agents, which are circulating at the gathering location, and because of the strain on the health care system of the host countries and communities. Infectious diseases outbreaks such as foodborne diseases as a result of suboptimum food and water hygiene, respiratory infectious diseases, and other infectious diseases associated with environmental conditions have been reported during MGs [3-5,8,9].

Government health care development plan of Iraq focuses on rebuilding the health infrastructure and developing its health care workforce, which has been damaged by instability in the country [10]. Thus, services for the Arbaeenia MG, including health care services, are not well developed, hampering the ability to abate the spread of pathogens and detect and respond to outbreaks in a timely manner. Routine health care services for the Iraqi population are inadequate because of ongoing conflicts within the country [11]. The annual Arbaeenia MG further burdens the national health system, during which attendees and local communities lack adequate access to health services.

In 2014, more than 19 million people from 40 countries of the world participated in this occasion, making it the second largest gathering in the world [12]. Although this MG has complex challenges and is associated with high burden of diseases, there have been few studies on the burden of religious MGs on local health resources of Iraq. The growing number of individuals who attend the event annually, the changing dates of the anniversary, and the short duration of the event highlight the importance of preparedness plans and resources to effectively manage the gathering by national and local authorities.

### Objectives

Some rudimental health care services, such as temporary clinics and services supported by faith-based groups, are offered to the MG attendees during the journey toward Karbala. This study aimed to assess the availability of medical supplies, equipment, medical instruments, and supplies for infection control at the temporary clinics in Al-Karkh, Baghdad, Iraq. The findings of this study are expected to reflect the level of preparedness for such MGs in term of resources availability. This would assist the Ministry of Health (MOH) in Iraq to be better prepared for the next event.

## Methods

### Design

A cross-sectional study was conducted on temporary clinics that serve the masses walking from Baghdad to Karbala through Al-Karkh and the surrounding areas. The temporary clinics were set up by the MOH; Ministry of Interior (MOI); Ministry of Defense (MOD); nongovernmental organizations (NGOs), such as the International Red Crescent Society; and faith-based civil society organizations, locally known as *mawakib*.

The temporary clinics were set up along the road that goes through Al-Karkh, Baghdad, toward Karbala city, a distance approximately 100 km long. The masses making the trip to the Arbaeenia MG in Karbala from Baghdad and nearby provinces use this road.

### Data Collection

We developed a checklist to collect the following information: clinic name, location, and affiliation (governmental or nongovernmental); availability of safe drinking water source, regular electricity, and air-conditioning system; type of building and number and category of health care workers in the clinic; and availability of basic medical equipment and instruments, drugs, and other medical supplies (developed based on the essential list of supplies determined by MOH for basic health units). The original list was acquired through personal communication with Baghdad Al-Karkh Directorate. Information on the average daily number of patients seen by the clinic was also collected.

The data were collected from December 6, 2014 (6 days before the Arbaeenia commences), and completed on December 13, 2014. The data collectors interviewed the clinic managers to complete the checklist.

### Data Analysis

Statistical Package for Social Sciences version 18 (Chicago: SPSS Inc) was used for data entry and analysis. We categorized the clinics into MOH and non-MOH clinics and generated the percent distribution of clinic attributes, category of health workers, availability of basic medical equipment and drug supplies, average number of patients served per day, and availability of supplies for infection control by clinic affiliation. Data were described using percentages and means.

## Results

### Clinics’ Characteristics

A total of 30 temporary clinics were set up along the road in Al-Karkh, Baghdad. Of these clinics, 60% (18/30) were set up by the MOH, 12% (5/30) by the MOI/MOD, and 23% (7/30) by NGOs. Almost two-thirds of visitors who attended these clinics were from Iraq; 15% from Iran; and small percentages from Bahrain, Lebanon, Saudi Arabia, Kuwait, Pakistan, India, Oman, Afghanistan, and other countries. More than three-fourths of the patients were aged between 20 and 59 years.

Of the temporary clinics, 15 were caravans, 11 were tents, and 3 were ambulances (Table 1). The MOH clinics were predominantly caravans, whereas the non-MOH clinics were predominantly tents. Of the 30 clinics, 25 had regular electricity and 16 had clean drinking water supply. Moreover, 12 clinics had air-conditioning, of which all were MOH clinics.

**Table 1:** Distribution of temporary clinics by the type of construction and availability of basic requirements.

Variable		MOH^a^ clinics (n=18), n (%)	Non-MOH clinics (n=12), n (%)	Total (N=30), n (%)
**Type of building**				
	Caravan	14 (77)	1 (8)	15 (50)
	Tent	2 (11)	9 (75)	11 (36)
	Concrete building	1 (5)	0 (0)	1 (3)
	Ambulance auto	1 (5)	2 (16)	3 (10)
**Available services**				
	Clean drinking water supply	10 (55)	6 (50)	16 (53)
	Air-conditioning	12 (66)	0 (0)	12 (40)
	Regular electricity	17 (94)	8 (66)	25 (83)

^a^MOH: Ministry of Health.

There were 61 workers including 26 paramedics, 18 physicians, and 17 security guards. The workers were predominantly males (67%). On average, a total of 6720 and 9485 patients had attended the MOH and non-MOH clinics, respectively. The average daily attendance per clinic was 540, and the average daily patients seen per health worker was 368.

### Basic Medical Equipment/Supplies

Both MOH-affiliated and non-MOH–affiliated clinics had a sphygmomanometer and a stethoscope, and 87% (26/30) of the clinics had a glucometer. All other medical equipments were available in 30% to 73% of the clinics (Table 2).

**Table 2:** Distribution of Ministry of Health and non–Ministry of Health temporary clinics by availability of basic medical equipment/supplies.

Variable		MOH^a^ clinics (n=18), n (%)	Non-MOH clinics (n=12), n (%)	Total (N=30), n (%)
**Diagnostic equipment/supplies**				
	Sphygmomanometer	18 (100)	12 (100)	30 (100)
	Stethoscope	18 (100)	12 (100)	30 (100)
	Glucometer	16 (88)	10 (83)	26 (86)
	Torch for mouth examination	9 (50)	0 (0)	9 (30)
	Tongue depressor	16 (88)	0 (0)	16 (53)
	Thermometer	17 (94)	2 (16)	19 (63)
	Examination bed (couch)	17 (94)	5 (41)	22 (73)
**Oxygen supply**				
	Nebulizer	15 (83)	2 (16)	17 (56)
	Oxygen cylinder	13 (72)	5 (41)	18 (60)
	Oxygen giving set	13 (72)	4 (33)	17 (56)

^a^MOH: Ministry of Health.

### Essential Medicines

All clinics had almost all the essential medicines, which included painkillers, antibiotics, antipyretics, antihistamines, ointments, hydrocortisone, and antidiarrheal drugs, with a range of 87% to 100% (Table 3). Drugs for diabetes mellitus and hypertension, ophthalmic ointments and drops, and fluids were the least available in the clinics (<54%). Only 17% (5/30) of the clinics had all basic drugs. The MOH-affiliated clinics had higher or similar availability of all drugs compared with the non-MOH clinics.

**Table 3:** Distribution of Ministry of Health and non–Ministry of Health temporary clinics by availability of basic drugs.

Drugs	MOH^a^ clinics (n=18), n (%)	Non-MOH clinics (n=12), n (%)	Total (N=30), n (%)
Painkiller	18 (100)	12 (100)	30 (100)
Antibiotics	18 (100)	8 (66)	26 (86)
Antipyretics	18 (100)	11 (91)	29 (96)
Antihistamine	18 (100)	11 (91)	29 (96)
Antihypertensive	15 (83)	2 (16)	17 (56)
Diabetes mellitus drugs	12 (66)	1 (8)	13 (43)
Ointments	16 (88)	19 (83)	26 (86)
Antidiarrheal	16 (88)	12 (100)	28 (93)
Ophthalmic drops and ointments	11 (61)	4 (33)	15 (50)
Distilled water for injection	17 (94)	7 (58)	24 (80)
Hydrocortisone injection	18 (100)	8 (66)	26 (86)
Fluids	13 (72)	3 (25)	16 (53)

^a^MOH: Ministry of Health.

### Basic Supplies for Infection Control

Almost all clinics had personal hygiene supplies, except alcohol-based handrub, which was available in approximately 60% of the clinics (Table 4). The medical waste disposal supplies were lacking in most clinics, and less than 50% of the clinics had medical waste supplies. Environmental sanitation supplies were available in 77% of the clinics; however, only 22% of all clinics had all infection control supplies.

**Table 4:** Distribution of temporary clinics by basic supplies for infection control.

Supplies for infection control	MOH^a^ clinics (n=18), n (%)	Non-MOH clinics (n=12), n (%)	Total (N=30), n (%)
Hand washing soap/liquid soap	16 (88)	11 (91)	27 (90)
Alcohol-based handrub	12 (66)	5 (41)	17 (56)
Disposable latex gloves	17 (94)	9 (75)	26 (86)
Disposable syringes	17 (94)	10 (83)	27 (90)
Receptacle (pedal pin) with lid and plastic bin liner	7 (38)	0 (0)	7 (23)
Sharp container safety box	14 (77)	0 (0)	14 (46)
Environmental disinfectant (chlorine or alcohol)	16 (88)	7 (58)	23 (76)

^a^MOH: Ministry of Health.

### Medical Supplies and Instruments

Equipment and supplies for cleaning and dressing wounds and injuries were available in almost all clinics, both MOH and non-MOH clinics. Of the 30 clinics assessed, only 4 clinics had equipment for instrument sterilization (Table 5).

**Table 5:** Distribution of Ministry of Health and non–Ministry of Health temporary clinics by availability of essential medical supplies and instruments.

Essential medical supplies and instruments	MOH^a^ clinics (n=18), n (%)	Non-MOH clinics (n=12), n (%)	Total (N=30), n (%)
Cotton and wraps	17 (94)	12 (100)	29 (97)
Bandages	17 (94)	12 (100)	29 (97)
Disinfectant and detergent	17 (94)	12 (100)	29 (97)
Autoclave	4 (22)	0 (0)	4 (13)
Surgical instrument for wound suturing	13 (72)	4 (33)	17 (57)

^a^MOH: Ministry of Health.

## Discussion

This assessment focused solely on the availability of medical supplies, medical equipment, and instruments for personal medical services, emergencies, nonemergency health problems, and infection control at the clinic level. The assessment of temporary clinics serving the masses walking toward Karbala from Al-Karkh, Baghdad, and surrounding areas showed that the supplies and manpower in the clinics demonstrated some capacity to handle minor common ailments encountered during the Arbaeenia MG. MOH clinics were more equipped with diagnostic equipment/supplies than non-MOH clinics. This reflects poor planning and coordination of services and highlights the need for coordination between MOH and other ministries and NGOs to ensure the availability of supplies and equipment in all clinics. The public health sector’s preparedness for MGs requires a huge investment of time, organization and intersectoral collaboration, planning epidemiological surveillance, organization of control and prevention measures, organization of health care services, and management of cases and casualties.

The drug supplies found in the clinics were useful for several ailments: (1) nonspecific symptom relief such as pain, fever, diarrhea, eye problems, and allergies; (2) antibiotic-treatable infections; (3) dehydration; and (4) chronic diseases (eg, diabetes mellitus and hypertension). Medical instruments needed to clean and dress wounds and injuries were available in the majority of clinics. Such instruments are much needed during such MGs because injuries are common during the MG [13]. Autoclaves for sterilizing surgical instruments were rare. Poor availability of sterilizing equipment might increase the hazards of infections. The MOH of Iraq should ensure the availability of such equipment in all clinics providing health services.

The health professionals in the clinics were physicians and paramedics. The ratio of paramedics to the physicians in the clinics assessed was 1.4:1, which is close to the ratio in Iraq (1.8:1) [14]. One-tenth of temporary clinics were ambulances. The capacity of the clinics and the number of health professionals were not adequate to serve the participants of the MG, as indicated by the average number of persons per clinic and per staff. The portion of the participants traveling from Al-Karkh, Baghdad, to Karbala in of need ambulance services was not known, and thus, the adequacy of ambulance service and its distribution along the road was not studied and could not be determined in this assessment.

The infection control materials for personal hygiene and control of infection and environmental sanitation were mostly available at the clinics. Medical waste disposal materials were rarely available at the clinics, which could pose a risk of infection to the patients at the majority of temporary clinics. The WHO Epidemic and Pandemic Alert and Response document [15] can be used by the MOH of Iraq to guide those responsible for the health needs of individuals attending an MG and to help them plan their actions.

The environmental conditions, such as safe drinking water supply, air-conditioning, and electricity, were mostly adequate, particularly for the MOH clinics. The non-MOH clinics were not equipped with air conditioners, which are important for the health and safety of patients, given the desert environment. Water and food safety and compliance with health conditions should be ensured before and during the event. Food handlers should be trained and provided with working guidelines for safe food handling, and their practices should be inspected and monitored during the event.

The priority during MGs is often to manage emergency and nonemergency personal medical problems and injuries of the attendees. The importance of safeguarding the larger global population from outbreaks of infectious diseases becomes a secondary priority during the MG. The Arbaeenia MG is becoming increasingly populous, and the government is not well prepared for managing imminent health emergencies and mass causalities. This assessment shows that various government ministries were involved in providing services to the masses walking to Karbala for the religious event, demonstrating a commitment from the Government of Iraq to address the needs of MG participants. Although the services were mainly medical services, some form of public health services accompanied the medical services, including personal hygiene and infection control supplies and medical waste disposal supplies. The public health impact of such services needs to be assessed in the future.

During MG, the health planners should consider the provision of care that is consistent with local standards of care for participants; meanwhile, they have to ensure the availability of continuing medical service to the populations surrounding the event venue and be vigilant and well prepared to respond to unusual events.

The MG in Karbala requires prior national planning, which the government understands, as demonstrated by its level of involvement. However, Iraq is undergoing continuous violence and war and is therefore limited in its capacity to plan for and provide adequate services to MG participants to protect the local and global populations from the spread of infectious diseases.

In conclusion, although temporary clinics were relatively equipped with basic medical supplies, equipment, and instruments for personal medical services, the health workforce was insufficient, given the number of individuals seeking care, and only limited public health service, personal infection control, and supplies were available at the clinics. This assessment should assist the Government of Iraq to develop an adequate plan for services at the annual Arbaeenia MG to develop rigorous national standards for provision of medical care during MGs. WHO’s global health initiatives and key considerations [3] should be considered in the process of setting up and implementing public health alert, response, and operational plans for MGs. These initiatives provide advice about prevention, detection, and management of public health incidents as well as the integration of the full range of public health activities into the MG planning process.

## Abbreviations

MG: mass gathering
MOD: Ministry of Defense
MOH: Ministry of Health
MOI: Ministry of Interior
WHO: World Health Organization
